# Polymeric Materials as Indispensable Tools to Fight RNA Viruses: SARS-CoV-2 and Influenza A

**DOI:** 10.3390/bioengineering9120816

**Published:** 2022-12-18

**Authors:** Ariana C. F. Santos, Fátima Martel, Carmen S. R. Freire, Bárbara J. M. L. Ferreira

**Affiliations:** 1 CICECO-Aveiro Institute of Materials, Department of Chemistry, University of Aveiro, 3810-193 Aveiro, Portugal; 2 Biochemistry Unit, Biomedicine Department, Faculty of Medicine, University of Porto, 4200-319 Porto, Portugal; 3 I3S-Institute of Research and Innovation in Health, University of Porto, 4200-135 Porto, Portugal

**Keywords:** RNA viruses, COVID-19, influenza A, polymeric materials

## Abstract

Towards the end of 2019 in Wuhan, suspicions of a new dangerous virus circulating in the air began to arise. It was the start of the world pandemic coronavirus disease 2019 (COVID-19). Since then, considerable research data and review papers about this virus have been published. Hundreds of researchers have shared their work in order to achieve a better comprehension of this disease, all with the common goal of overcoming this pandemic. The coronavirus is structurally similar to influenza A. Both are RNA viruses and normally associated with comparable infection symptoms. In this review, different case studies targeting polymeric materials were appraised to highlight them as an indispensable tool to fight these RNA viruses. In particular, the main focus was how polymeric materials, and their versatile features could be applied in different stages of viral disease, i.e., in protection, detection and treatment.

## 1. Introduction

**Figure 1:**
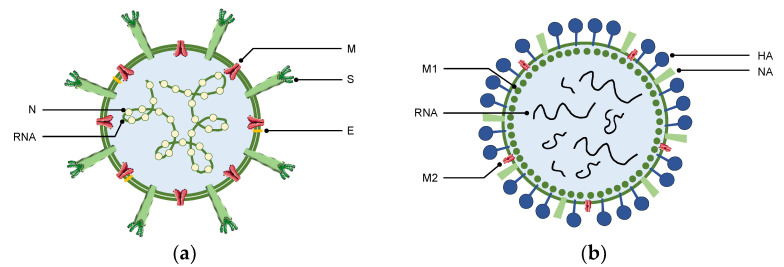
Schematic representation of (**a**) the coronavirus and its structural proteins (envelope (E), membrane (M), nucleocapsid (N) and spike surface glycoprotein (S)); and of (**b**) Influenza A and its membrane proteins (hemagglutinin (HA), neuraminidase (NA) and matrix (M)).

Coronaviruses (CoV) are a family of viruses called *Coronaviridae* that may be divided into four groups: α-, β-, γ- and δ-coronavirus [1,2]. In particular, severe acute respiratory coronavirus 2 (SARS-CoV-2) that causes COVID-19, is a type of β-CoV responsible for acute respiratory tract infections [3,4]. Coronaviruses have four major structural proteins in their structure, namely, envelope (E), membrane (M), nucleocapsid (N) and spike surface glycoprotein (S) [5,6], as illustrated in Figure 1. It is through the binding of S proteins to cellular receptors that SARS-CoV-2 enters the host cells. After fusion with the host endosome membrane, the S protein is recognized by angiotensin-converting enzyme 2 (ACE2) receptors and there is a cleavage of the trimer S protein (by the cell surface-associated transmembrane protease serine 2, TMPRSS2) and of cathepsin [7]. The SARS-CoV-2 envelope fuses with the endosome membrane in the lysosomal acidic environment and it occurs the release of the genetic material (viral RNA) into the host cell. The latter wrongly recognizes the viral RNA as its own and SARS-CoV-2 undergoes viral RNA replication within the host cells [8,9]. Viral E, M, N and S proteins are also translated in the endoplasmic reticulum and Golgi apparatus, thus allowing the packaging of new viruses. The assembled viruses are then released via exocytosis into the extracellular compartment to begin a new (infectious) life cycle [2,10].

SARS-CoV-2 (COVID-19) is very similar to the influenza virus (flu). In fact, both are RNA viruses and present equivalent infectivity characteristics, including high incidence, rapid onset and easy mutation [11,12]. Flu replication also occurs in the respiratory tract epithelium with the production of viral proteins [11]. Influenza virus is an enveloped virus of the *Orthomyxoviridae* family and is classified into four genera, namely influenza A (IAV), B (IBV), C (ICV) and D (IDV) viruses. IAV, the most common type, is further subtyped by two surface glycoproteins located within the host-derived lipid membrane of virions, hemagglutinin (HA) and neuraminidase (NA) [13,14]. Therefore, the specific name of the virus is given based on the proteins it expresses, i.e., the standard nomenclature is influenza A HxNx, where the “x” is the number of specific types of HA and NA [15].

Usually, IAV has three membrane proteins: NA, HA, and the matrix 2 (M2) [16]. The matrix 1 (M1) is right under the membrane and is surrounded by eight viral RNA (Figure 1). M1 has an important role for cell membrane binding, whereas HA is the contributing factor in IAV infection [17]. In other words, the HA protein binds to a host receptor with sialic acid and glycolipid receptors and enters the cell by endocytosis. During this process, the encapsulated viral RNA is released and the consequent transcription and replication takes place [11]. However, and despite all the similarities between SARS-CoV-2 and influenza A, COVID-19 is still a more serious illness for some people compared to flu, according to the Center for Disease Control and Prevention (CDC) [18]. COVID-19 can also take longer before the infected individuals show the first symptoms. While COVID-19 symptoms may appear 3 to 7 days after the initial exposure to the virus, patients with influenza start to abruptly feel the first signs of infection after 2 to 5 days [19]. So, people infected with SARS-CoV-2 can remain contagious for longer periods, resulting on an easier propagation of the virus. In this context, it is important not only to pay attention to viral-infection symptoms, but also to test regularly to proceed to an early disease diagnose and avoid COVID-19 spread.

### Flu or Corona? Maybe Flurona

The transmission of influenza and SARS-CoV-2 viruses occurs through respiratory droplets and these droplets can stay either in the nose or mouth or enter the lungs via the inhaled air [1,20,21]. In both cases, the most common symptoms are dry cough, fever, fatigue, muscle pain, headache, diarrhea and dyspnea [2,3]. However, people with chronic comorbidities, such as hypertension, diabetes, coronary heart disease, cerebral infarction, chronic bronchitis, asthma, among others, are more likely to develop more serious complications, including fatality. For COVID-19 infection, male adults seem to be more affected than females, although not every person develops symptoms when they contract the virus [3]. Protection should then be considered for all people.

It may not be easy to diagnose acute respiratory infections, as a wide range of pathogens can cause similar clinical syndromes [22]. It is even harder to identify (and treat) a simultaneous infection caused by SARS-CoV-2 and influenza, i.e., flurona [23]. Additionally, several studies have shown that viral co-infections are associated with disease severity, acute respiratory distress syndrome (ARDS) and even death [24]. Although the percentage of hospitalized flurona patients is relatively low, it has been a growing concern among public health experts, especially because these hospitalizations were highest in January 2022 compared to all the previous months of the pandemic [25].

In order to avoid this viral storm, specific assays to screen and detect both SARS-CoV-2 and influenza are recommended for all suspected patients [19]. For that, serological and molecular approaches are available. Serological tests do not identify the virus but do provide information on the type and concentration levels of various immunoglobulins (IgA, IgM and IgG) that are produced during the infection. Serological testing evaluates the immune response mediated by antibodies, so the accuracy of the test results still remains challenging [26]. On the other hand, molecular diagnosis focuses on nucleic acid testing [27]. The standard approaches include gene sequencing, nucleic acid amplification (e.g., polymerase chain reaction, PCR) and clustered regularly interspaced short palindromic repeats (CRISPR) [27]. Among these, the real-time polymerase chain reaction (RT-PCR) test is the preferable method to detect unique sequences of the viral RNA [1]. The genes that may be identified are the N, E and S proteins as well as the RNA-dependent RNA polymerase (RdRP) genes for SARS-CoV-2 [28], and the NA and M genes for influenza [29]. Of course, this technique has some drawbacks, namely its sensitivity and specificity. Sometimes, RT-PCR tends to give false-positive and false-negative results. For COVID-19, a false-negative result may be correlated to deletions and mutations in the SARS-CoV-2 genome, which tend to occur during evolution, or due to a possible co-infection (with influenza) [30]. Nevertheless, since RNA-based methods are expensive and time-consuming [1], other sensing strategies are being developed for viral detection as they can possess better sensitivity, selectivity, specificity, authenticity and scalability [31,32].

The next step (after the viral detection) is the treatment. The symptoms of COVID-19 and flu can be reduced with the use of antibodies or antiviral drugs [33]. However, recent approaches with the use of polymeric materials can be explored as good alternatives to help fighting these airborne viruses. In this context, this review aims to put together the most recent options that exploits polymeric materials for individual protection, detection, and treatment against both SARS-CoV-2 and influenza A. More sustainable options, considering the use of biopolymers instead of synthetic ones, will be also highlighted as a more advantageous way to overcome these diseases. Finally, future perspectives on this matter will be discussed.

## 2. Protection

**Figure 2:**
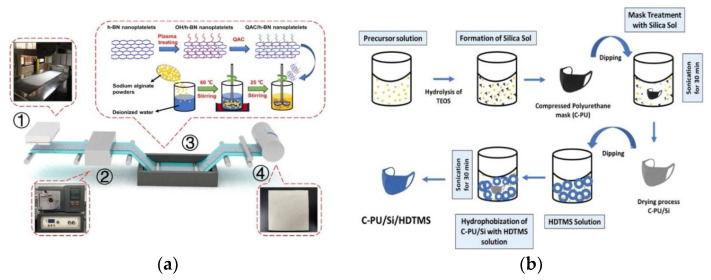
(**a**) Scheme of the preparation process of the QAC/h-BN/PP nanocomposite fibrous membranes. The preparation process includes four steps as follows: (1) preparation of PP ultrafine fiber nonwovens by melt-blown processing with high-speed and high-temperature hot wind. (2) Treatment of the meltblown PP ultrafine fiber nonwovens with oxygen plasma to activate their surface. (3) Immobilization of quaternary ammonium salt (QAC) organic chains, with extended-spectrum antibacterial activities, on the surface of hydroxylated h-BN nanoparticles (through the formation of covalent bonds) and consequent immersion of the activated PP ultrafine fiber nonwovens in the QAC/h-BN nanoplatelet suspension. (4) Drying and hot-pression of wet QAC/hBN/PP composite nonwovens to obtain QAC/h-BN/PP nanocomposite fibrous membranes. (**b**) Treatment process of silica sol and hydrolyzed hexadecyltrimethoxysilane (HDTMS) to produce self-cleaning, anti-droplet masks. (Reprinted with permission from [40] and [41]. Copyright American Chemical Society, 2020 and Elsevier, 2020).

As it is well-known, the basic safety measures include vaccination, maintaining a social distance while avoiding poorly ventilated spaces, effectively disinfect surfaces, correct hand hygiene, cover coughs and sneezes, test regularly and, of course, use protective equipment [34]. According to the World Health Organization (WHO), protective equipment may include face masks, gloves, goggles, face shields and gowns [35]. Face masks have been identified as the most effective respiratory protective device, since they act as a physical barrier to the respiratory droplets and reduce viral transmission between individuals [36,37]. Additionally, face masks can be divided into respirators and surgical masks. Respirators are well-fitted devices that can prevent the inhalation of particles that may cause respiratory infection and be harmful to the wearers. Surgical face masks do not provide full protection from inhalation of viruses (or other airborne pathogens), but work as a fluid-resistant barrier to body fluids or droplets, being able to reduce the contamination between persons [36,38]. Usually, these face masks are made of synthetic polymers, such as polypropylene (PP), polyurethane, polyacrylonitrile or polystyrene [39]. Recently, Xiong and co-workers reported a PP nanocomposite face mask with excellent comfortability and antibacterial activity [40]. The mask was composed of melt-blown PP ultrafine fiber nonwovens, functionalized with hexagonal boron nitride (h-BN) nanoparticles, quaternary ammonium compounds (QAC), and a conductive inorganic (nano)material. The procedure used to prepare these masks is summarized in Figure 2a. The achieved results showed that this fiber membrane-based mask exhibited both thermal comfort and antibacterial performance. In particular, the thermal conductivity of commercial PP nonwovens was only 0.13 W m^–1^ K^–1^, whereas the values obtained for the QAC/h-BN/PP nanocomposite fibrous membranes reached 0.88 W m^–1^ K^–1^. This represented an enhancement in the thermal conductivity of 706.5% comparing to commercial PP nonwovens. These QAC/h-BN/PP masks also displayed more substantial heat dissipation (could reach a surface temperature of 33.6 °C when used for 60 min) than commercial PP surgical masks (reached 31.8 °C during the same time). Regarding the antibacterial tests, the QAC/h-BN/PP nanocomposite fibrous membranes were able to kill 99.3% and 96.1% of *E. coli* and *S. aureus*, respectively, through “contact killing” mechanisms, i.e., QAC/h-BN/PP nanocomposite fibers killed bacteria only when they came into contact with bacteria [40]. In a different vein, Ray and co-workers developed an anti-droplet and hydrophobic coating for application on compressed-polyurethane (C-PU) masks. The coating was achieved by using silica sol and hydrolyzed hexadecyltrimethoxysilane (HDTMS), as presented in Figure 2b. The resulting modified mask (C-PU/Si/HDTMS) demonstrated good water repellency, achieving a higher water resistance (water contact angle of 132°) for a longer period of time when compared to the unmodified mask (85°). Additionally, due to the low sliding angle of the inclined surface, the modified mask exhibited a self-cleaning ability. Therefore, the proposed coating for C-PU masks may have great potential in controlling viral infection [41]. In a different study, Xiong and colleagues prepared superhydrophobic and superoleophobic composite nanofiber membranes to use as a filter element in protective masks [42]. These composite nanofibers were successfully prepared through the modification of polyacrylonitrile (PAN) by fluoro-polyurethane (FPU) doping. The PAN/FPU nanofiber membrane, prepared by electrospinning, had an excellent surface stability since it maintained its initial contact angle after 240 s, contrasting with PAN nanofiber membranes that reached 0° after the same time. The quality factor (Qf) of the PAN/FPU nanofibers, i.e., the filtration performance of these materials or, in other words, the removal capability of oil particulate pollutants in masks, was much higher than those of commercial masks by over 50%. Consequently, the developed PAN/FPU nanofibers are effective and have good market prospects as protective filtration materials [42]. Li et al., prepared hybrid electret fibers based on polystyrene/poly(vinylidene fluoride) by studying the complementarity of electric responses between polystyrene (PS) and polyvinylidene fluoride (PVDF) [43]. The coupling of the electric polarization behaviors of these polymers allowed an enhanced electret effect and high porosity of the hybrid PS/PVDF fibers. Therefore, the protective respirator using PS/PVDF fibers as the core layer exhibited high filtration efficiency (99.752% compared with other reported air filters, such as polyvinylidene fluoride nanofibers hybridized by polytetrafluoroethylene nanoparticles [44] or ultrathin poly(vinylidenefluoride-co-trifluoroethylene) (PVDF-TrFE) nanofibers) [45], as well as low air resistance (72 Pa, representing only 1/5 of the maximum limit ~320 Pa in the national institute for occupational safety and health, NIOSH, standard) [43].

Besides face masks, other protective equipment was developed to achieve extra protection towards SARS-CoV-2 or other respiratory viruses. For instances, Karagoz and co-workers designed a multifunctional material for protective clothing applications. Specifically, flexible electrospun poly(methyl methacrylate) (PMMA) nanofibers were decorated with ZnO nanorods and Ag nanoparticles (PMMA/ZnO-Ag NFs). This novel material showed high performance as: (i) an antibacterial agent (against both *E. coli* and *S. aureus* with their respective diameter zones of inhibition of 7–17 and 8.5–18.5 mm), (ii) an antiviral agent (inhibition of coronavirus, BCV, and parainfluenza viruses, BPIV3 with values of 3.75 and 1.75, respectively, for 1 h; and 4.25 and 4, respectively, for 24 h), (iii) a photocatalyst (capable of degrading organic pollutants, in particular, 75 and 44.5% for PMMA/ZnO and PMMA/Ag, respectively, after 300 min) and (iv) a reusable surface-enhanced Raman scattering (SERS) substrate (for the quantitative analysis of trace pollutants on the nanofiber with concentrations ranging from 100 μM to 1 nM. The characteristic peak of methylene blue at 1625 cm^−1^ was observed at concentrations as low as 1 nM) [46]. Another example is the work conducted by Khanzada et al., where aloe vera and polyvinyl alcohol (AV/PVA) electrospun nanofibers with antibacterial applications were developed [47]. The authors were the first to explore the antibacterial activities of AV/PVA and they claimed that these fibers could be suited for the preparation of protective clothes to use against COVID-19. For the possible designing of reusable and re-sterilizable personal protective equipment, Cuthbert and co-workers described the production of a polypropylene-based nonwoven filter, which was covalently functionalized with a zinc–porphyrin photosensitizer using diazirine C–H activation chemistry, and its potential antiviral activity was evaluated against influenza A [48]. Covalent attachment approaches have the advantage of reducing leaching, i.e., the possibility of releasing the active components (otherwise, there would be an inevitable decrease in performance over time). Additionally, covalent attachment of photosensitizers allows the surface of a material to be modified without affecting the bulk properties. The C-H activation strategy was thus employed during this process to allow the covalent functionalization of nonwoven melt-blown polypropylene textiles with a common photoactive molecule (zinc-porphyrin). In this work, the photosensitizer-functionalized polypropylene filter was tested against IAV over a period of four hours with high intensity visible light and it proved to inactivate the virus by 99.99% comparing to the control [48].

Another study with hopeful applicability to COVID-19 or flu was carried by Sun and co-workers. In this work, they prepared daylight active functional polymeric nanofibers membranes containing vitamin K (VNFMs) [49]. The authors aimed that these membranes could serve as antiviral and antibacterial materials for face masks or other personal protective equipment. Vitamin K is known for being a photoactive chemical, derived from natural products, with the ability of producing reactive oxygen species (ROS). Daylight active functional polymers, namely polyacrylonitrile and poly(vinyl alcohol-co-ethylene), were used as a matrix and blended with vitamin K. The electrospinning technique was employed to obtain nanofibrous membranes in a letter size. The VNFMs exhibited photoactivity by generating ROS under both daylight (D65, 300−800 nm) and ultraviolet A (UVA, 365 nm) irradiation, resulting in high antimicrobial and antiviral efficiency (>99.9%) against *E. coli*, *L. innocua*, T7 bacteriophage and infectious peritonitis within a short exposure time (<90 min). In particular, under the same photoirradiation, poly(vinyl al*co*hol-*co*-ethylene)-based membranes (PVA-*co*-PE) produced more ROS and showed better antibacterial and antiviral properties when compared to polyacrylonitrile-based nanofibers, indicating that PVA-*co*-PE was a better matrix polymer material for these functions [49]. However, this was a preliminary study and further assays are needed to understand the potential of these membranes as inhibitors of SARS-CoV-2 or IAV.

Although effective, the previously described examples had different synthetic non-biodegradable polymeric materials in their composition, which contributes towards environmental pollution [39,50]. Particularly, the widespread use of face masks and their consequent disposal along the current pandemic can negatively affect and impact both human and animal health. Therefore, more sustainable options should be considered in the preparation of these protective medical devices. In this line, Deng and co-workers reported an easy and potentially scalable method to fabricate biodegradable, breathable, and biocidal cellulose nonwovens (BCNWs) to address both environmental and hygienic problems of commercially available face masks. In their work, they covalently grafted highly biocidal agents, namely polyhexamethylene guanidine (PHG) and neomycin sulfate (NEO), on cellulose nonwovens (BCNWs). These BCNWs proved to rapidly inactivate >99% of the SARS-CoV-2 and HCoV-229E viruses, as well as Gram-positive and Gram-negative bacteria after a 30-min and 15-min contact time, respectively [51]. Cellulose is the most abundant biopolymer in the world. The properties of this natural polymer go from high strength to low toxicity and its transformation into nanocellulose can be advantageous for the fabrication of mask filters. In particular, nanocellulose masks can have a filtration efficiency up to 99.9995%, [52] making this biopolymer a desirable and appropriate material for future face mask production with a robust bio-protection, without compromising the interception efficiency and the negative impact on the environment [51]. The surface chemical modification of nonwoven cellulosic fiber filters can thus improve the antiviral properties of face masks, in particular against respiratory influenza A virus [53]. Tiliket and co-workers modified nonwoven cellulosic fiber filters with poly(ethylenimine) (PEI), a synthetic, non-biodegradable and cationic polymer with amine functional groups [54]. Due to the high content of protonable amine groups, PEI is an attractive candidate to impart positive charges onto the surface of fibers and capture viruses through physicochemical interactions [53]. Just by chemically modifying the cellulosic fibers of the commercial medical masks, by fixing PEI, a remarkable improvement of the affinity for airborne viruses was obtained and the modified medical mask proved to block droplet-borne/airborne IAV. In addition, the modified masks allowed the removal of 99.999% of a sprayed solution of T4D bacteriophages after 1 h [53]. Another study conducted by Catel-Ferreira and co-workers also aimed to modify the surface of commercial nonwoven cellulose supports with catechin [55]. There are studies in the literature that highlight the strong antiviral activity of catechin (e.g., extracted from green tea) towards influenza viruses [56]. In particular, this polyphenol is able to inhibit HA and NA activities and leads to a high dose-dependent suppression of viral RNA synthesis together with an alteration of viral membrane physical properties. Therefore, its incorporation into masks to introduce antimicrobial properties was explored. Virus filtration experiments were performed by spraying an aerial suspension of T4D bacteriophage virus for different times. The two layers of catechin-functionalized filter had the best virus capture factor, *f*, of 2.9 × 10^3^ and a removal of 5-log of the sprayed bacterial solution after 2 h. This improvement in the reduction in the viral concentration in liquid media encouraged the authors to suggest this catechin-cellulose based-system as an eco-friendly filter for air purification [55]. Nevertheless, other biopolymeric materials can be considered for the development of viral protective equipment, namely sodium alginate. The latter is a polysaccharide extracted from brown seaweeds with chelating, immunogenic and mucoadhesive properties, making it an attractive polymer for drug delivery systems [57,58]. Since this natural polymer has interesting properties that may be applied in different fields, Bataglioli and co-workers developed a hybrid alginate–copper sulphate textile coating for disposable masks envisioning coronavirus inactivation [59]. Copper is a cheap and abundant trace element that is known for its antimicrobial and antiviral properties [60,61]. Two different methods were employed for coating deposition: immersion and multilayer coating. The fabrics coated with alginate–Cu(II) by the immersion procedure presented a 99% viral inactivation when in contact with the virus for less than 1 min. The multilayer deposition, in which the combination of biopolymer and metal ions occurred only in the fabric surface, enabled the use of higher concentrations of copper salt and biopolymer, leading to a viral inactivation of 99.99% at a contact time of 5 min without increasing cell toxicity or promoting copper–alginate agglomerates. The presence of alginate can improve the biocompatibility and adjust the metal ion availability in hybrid coatings. The authors suggested that the copper immobilization onto the fabric surface provided its fast release in aqueous medium and consequent interaction with the viral RNA, or its lipidic capsid, upon viral contact [59].

With the uncertainty and insecurity of becoming infected with SARS-CoV-2, people started to use disinfectants in their daily lives. However, the frequent use of disinfectants to clean surfaces is also not an ecofriendly or sustainable solution to control the spread of SARS-CoV-2 since they can not only affect the environment, but also have side effects on human/animal health (e.g., by forming poisonous or mutagenic secondary products in our bodies) [62,63]. With the help of nanotechnology, it is possible to have self-sanitizing surfaces by developing a permanent coating for daily materials or protective equipment, such as clothes or surgical dressings, beddings or wipes [64]. For instances, Hamounda and co-workers developed a cellulose-based wipe treated with silver nanoparticles (AgNPs). The antiviral activity of the AgNPs was evaluated against Middle East respiratory syndrome coronavirus (MERS-CoV) (also a β-coronavirus, similarly to SARS-CoV-2), proving to have good inhibitory effects. More precisely, comparing the number of plaque-forming units (PFUs) in sample-treated cells with the PFUs and in the untreated virus control cells, it was possible to determine a viral inhibition of 48.3% by the silver nanoparticles. Despite the reduced viral inhibition percentage (although still comparable with other cellulose-based wipes [55]), the authors claimed that these disinfectant wipes were effective and should be used in critical areas such as hospitals, healthcare centers or crowded places, as they seemed to reduce the risk of coronavirus infection [65]. Other approaches to develop a smart functional coating with long-term “release-killing” or “contact-killing” for IAV were explored by Li and co-workers [66]. In this study, a stabilized chlorine dioxide (ClO_2_) aqueous solution (anti-pathogen coating) was encapsulated in triblock copolymers of polyoxyethylene–polyoxypropylene. ClO_2_ is an approved antibacterial and antiviral agent that proved to have a slowly sustained-released from micelles. Copper NPs were also covalently clustered on these anti-pathogen micelles to improve contact-killing and micelle stability. The green synthesis of copper NPs involved the use of ascorbic acid, not only because ascorbic acid worked as a reducing and protecting agent, but also because it was able to increase the virucidal activity of copper [66]. The designed coating demonstrated a broad-spectrum of activity to kill drug-resistant bacteria, spores and viruses, in particular influenza A, in a short period of time. More specifically, the protein envelop of the H1N1 virus was damaged 1 min after coming into contact with the multi-functional coating. The authors believe that ClO_2_ mediated the oxidative damage of the virus, with the elimination of the hemagglutinin function [66].

Regarding vaccination as a safety measure, several COVID-19 vaccines using different platforms including nucleic acid-based vaccines (DNA or mRNA vaccines), adenovirus-based vaccines, protein-based vaccines, and inactivated vaccines have been introduced, among which many have received approval for prevention against COVID-19 [67]. Of these, the two vaccines that have shown the most promising results in preventing COVID-19 infection (the COVID-19 vaccines developed by Pfizer-BioNTech [68] and Spikevax [69]) are very similar in their formulation and represent a new class of vaccine products: they are composed of messenger ribonucleic acid (mRNA) strands encapsulated in lipid nanoparticles (LNPs) [70]. An advantageous characteristic of the nucleic acid-based vaccines, in relation to conventional vaccines, is the feasibility of their scale-up procedure and of industrial production in a short time, i.e., a quick development process. Another advantage of these vaccines relates to safety concerns of working with virulent viruses. However, they also possess some disadvantages, such as their low stability and their low affordability in low/middle-income countries, when compared with, e.g., viral vector vaccines [71].

Very briefly, both these mRNA-LNPs vaccines use mRNA that codes for the SARS-CoV-2 spike protein (S). The mRNA is encapsulated in a lipid nanoparticle (LNP) and after its entrance into the body by muscle injection, the generated S proteins are able to infect the host cells and the latter can produce spike antigens that will trigger an immune response. Accordingly, the body starts to produce antibodies and also forms memory cells [72,73,74]. The LNPs in mRNA COVID-19 vaccines consist of four main components, which are: a neutral phospholipid, cholesterol, a polyethylene-glycol (PEG)-lipid, and an ionizable cationic lipid [75]. In both cases, PEGylation has been employed in the vaccine formulation, since PEG facilitates the formation of a hydrophilic protective layer that stabilizes the vaccine lipid nanoparticles (avoids aggregation), improves the storage stability and decreases non-specific protein adsorption [75,76]. However, several allergic reactions to PEG in people who received Pfizer-BioNTech and Spikevax vaccines have been reported [76]. In this context, chitosan, a natural cationic polymer, may represent an alternative adjuvant for vaccine delivery for its well-known characteristics, such as biosafety, biocompatibility and mucosal adsorption-promoting (mucoadhesive) properties [77]. In this context, Jearanaiwitayakul and co-workers developed a potential intranasal vaccine candidate based on the incorporation of a receptor-binding domain (RBD) of SARS-CoV-2 spike glycoprotein (S) into N,N,N-trimethyl chitosan (TMC) nanoparticles (RBD-TMC NPs) [78]. Intranasal (IN) vaccines could represent a more advantageous way to induce long-lasting systemic and humoral immunity against mucosal pathogens, compared to intramuscular vaccines, since the latter can poorly control viral replication and nasal shedding in the upper respiratory tract [79,80]. The generated cationic NPs were easily deposited in the negatively charged mucosal layer, via ionic bonding, and promoted antigen uptake by the mucosal cells. The RBD-TMC NPs also stimulated a systemic humoral immune response, as revealed by the upregulation of circulating IgG, IgG1, IgG2a and IgA, as well as neutralizing antibodies. Overall, this vaccine platform was able to stimulate not only mucosal immunity and systemic humoral response (with a robust production of IgA and IgG in mice immunized with four doses of RBD-TMC NPs), but also a cell-mediated immune response (with the great development of CD8+ cells) in mice on day 45 after intranasal immunization [78]. Similarly, Liu and co-workers explored the potential of TMC nanoparticles, also for nasal administration, against influenza A. In this case, the authors conjugated an IAV antigen to the surface of TMC nanoparticles (through thioester bonds) in order to increase the immunogenicity of the antigen after nasal administration. The conjugation of the IAV antigen with these chitosan NPs (H1N1-TMC/NP), instead of its encapsulation into the TMC nanoparticles, resulted in an optimal immune response with the increased production of IL-1β (by macrophages) at 16 mg/mL as well as IL-2 (by lymphocytes) at 64 mg/mL [81]. These results showed that the mucoadhesive properties of TMC may be applied in the development of a promising nasal spray for human vaccination against viral infections. Nevertheless, other biopolymeric nanosystems can be considered potent adjuvants and delivery systems for viral threats, such as the previously mentioned sodium alginate [82].

In a study carried by Dehghan and co-workers, alginate particles, with sizes below 600 nm, were efficiently synthesized by the ionic gelation method, followed by the incorporation of influenza virus and adjuvants (either CpG oligodeoxynucleotide, CpG ODN, or quillaja saponin, QS) [83]. The humoral and cellular immune responses of the particles were evaluated in rabbit nostrils and, according to the obtained data, the combination of CpG ODN adjuvant with influenza virus resulted in a higher immunogenic potential, since it was able to activate B and dendritic cells and induce the secretion of cytokines, when compared to QS, which was not able to stimulate the immune system [83]. In a different manner, Boesteanu and co-workers proposed a safer, less-consuming dose and a faster way of delivering influenza vaccines by encapsulating live influenza viral strains in alginate hydrogels. The safety of subcutaneous vaccination was tested in mice and the authors concluded that the encapsulation of the virus in alginate preserved the antigenicity of the vaccine, as well as generated a robust T cell response. In fact, the results showed that the encapsulated live influenza delivered subcutaneously could trigger an efficient CD8+ T cell immune response in mice. On day 7 post challenge, the frequency of lung CD8+ cells was 26 ± 3% in mice that received the polymer-encapsulated vaccine, which was significantly higher comparing to mice injected with alginate alone (1.4 ± 0.07%) or non-vaccinated mice (2 ± 0.6%) [84].

Finally, to avoid the spread of COVID-19 and protect individuals from the virus, Moakes and co-workers formulated a nasal spray containing gellan gum and carrageenan [85]. The nasal passage represents a major player in the frontline defense, since it is responsible for filtering harmful microorganisms that are present in inhaled air. Therefore, the formulation of medicines and/or devices able to protect and control this area is very appreciated. Gellan gum is an anionic polysaccharide hydrogel-forming polymer with interesting characteristics, such as versatile textures, stability and biocompatibility [86,87]. Similarly, carrageenan is a biopolymer that typically forms highly viscous aqueous solutions and it is known for having antiviral activity against several enveloped viruses, including human immunodeficiency virus, herpes simplex virus, human cytomegalovirus, human rhinoviruses, among others [88]. The study aimed to develop a protective coating for the upper respiratory tract. For this, it was important to carefully choose a polymer that could increase spray lifetime. The mucoadhesive properties of both gellan gum and carrageenan allowed their adhesion to the mucosa, in particular the gellan gum systems proved to uniformly coat the nasal cavity, demonstrating high levels of coverage across the studied concentrations. In fact, the systems containing a greater proportion of gellan gum (75:25, gellan gum to λ-carrageenan) showed significant suppression of the infection up to a dilution of 1/100 in comparison with the untreated control group. This was unexpected since the gellan gum system itself showed limited ability to suppress the SARS-CoV-2 (contrary to λ-carrageenan that demonstrated complete inhibition over 48 h). It was hypothesized that the electrostatic charge distribution of the carrageenan within the solvent enabled the binding to the cellular membrane and when gellan gum is at a higher ratio it facilitates this role, leaving the carrageenan less hindered. The spray systems demonstrated high potent capacities to prevent SARS-CoV-2 infection in Vero cells, resulting in complete inhibition when either treating the cells or the virus, prior to challenging for infection [85]. An in-between strategy of treatment and protection was the one followed by Shrivastava and co-workers. In their work, they described the conception of a long-lasting (4–6 h), absorbent, osmotic, glycerol-based polymeric film using an in vitro nasal mucosa-mimicking model, containing polymers capable of not only cleaning the nasal surface, but also neutralizing pro-inflammatory cytokines and the COVID-19 S protein (Covispray) [89]. Cytokines are responsible for the activation, regulation and amplification of the immune response and their production is usually highly regulated in order to prevent systemic damage [90]. However, during viral infection, there may be significant pro-inflammatory cytokine expression, so-called “cytokine storm” (CS), which can result in inflammatory cell recruitment (associated with immune dysregulation, inflammation and hypercytokinemia [91]) and even lung tissue damage [92,93]. Therefore, this Covispray may be a promising approach to fight COVID-19. Different formulations were explored, but, in general, this spray contained glycerol (several percentages were tested), two jellifying agents, namely hydroxypropyl cellulose (HpP) and solagum (associated with acacia gum enrobed in xanthan gum) and natural compounds called S1 cyanidins (CsL, ClR, UdP, TpF). The latter (plant tannins) are big and inert molecules with a high affinity for pro-inflammatory cytokines. A concentration of 9.8% glycerol as an osmotic liquid with 0.30% HpP and 0.25% solagum as film jellifying ingredients, was selected for the conception of the initial Covispray film. This osmotic filmogen worked like a mask when applied in the nasal cavity, so the authors claimed that Covispray was not an antiviral or anti-inflammatory drug. Instead, the spray was considered as a protective layer that could be used to minimize the concentration of pathogens and as a multitarget treatment that could trigger an anti-inflammatory response. The Covispray’s final composition at a concentration as low as 5.0%, blocked on average 50.6% S1 protein and 49.6% RBD protein while at 10% concentration the inhibition was 72.2% and 72.0%, respectively. Furthermore, at 5% concentration, the final Covispray composition neutralized nearly 30% of IL-6, 80% of TNF-α and GM-CSF, as well as more than 90% of IL-10 and IL-13 (pro-inflammatory cytokines responsible for nasal and systemic inflammation). Thus, the authors considered Covispray an effective, safe and preventive treatment against multiple nasal pathologies, including COVID-19 positive symptomatic patients in early stages of the disease [89]. In Table 1, there is a summary of the different polymer-based approaches described in different studies in order to avoid viral contaminations and diseases.

## 3. Detection

**Figure 3:**
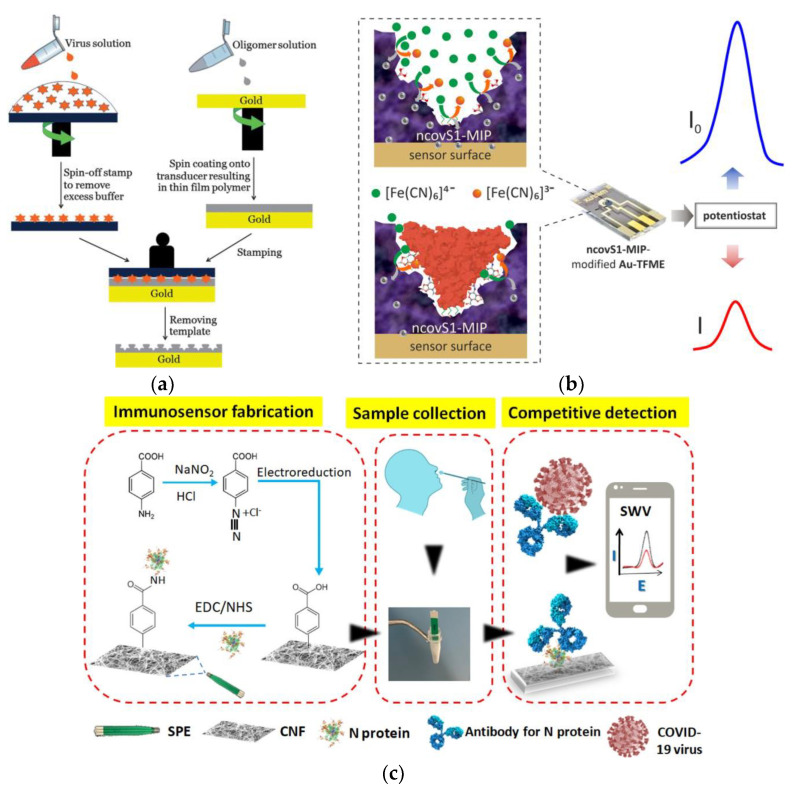
(**a**) Schematic representation of the imprinting protocol used to apply on the quartz crystal microbalance (QCM) as a biosensor. (Reprinted with permission from [97]. Copyright Royal Society of Chemistry, 2013). (**b**) The operating principle of the ncovS1 sensor in COVID-19 diagnosis, where the redox probe readily carries the charge through ncovS1-MIP to produce current (I0), and the rebound ncovS1 blocks pathways for the redox probe to carry the charge through ncovS1-MIP leading to a concentration-dependent contraction in the recorded current (I). (Reprinted with permission from [98]. Copyright Elsevier, 2022). (**c**) Schematic representation of the cotton-tipped electrochemical immunosensor for COVID-19. The sample collection is made by using the cotton-tipped electrode, the functionalization of the carbon nanofiber electrode is made by using electroreduction of diazonium salt and by attaching the virus antigen, and the detection principle is made by using a competition assay and the SWV technique. (Reprinted with permission from [99]. Copyright American Chemical Society, 2020).

Molecularly imprinted polymers (MIPs) are one example of electrochemical nanosensors that can be employed for the detection of SARS-CoV-2 and influenza viruses. MIPs have specific molecular recognition sites that are complementary to the shape and orientation of the targeted molecule, allowing a distinction between different molecules [94]. This method can be used as a template to design the specific targeting sequence in viruses. The molecule is exposed to functional monomers, which find their optimal position and are stabilized through a self-assembly process, and then the monomers are photopolymerized. Finally, the template is removed from the polymerized system, leaving behind an empty cavity that is called imprinting [95]. This technique was first applied by Wangchareansak and co-workers to screen influenza A viral subtypes [96,97]. To produce the MIP, they used acrylamide, methacrylic acid, methyl methacrylate, and N-vinylpyrrolidone as monomers and mixed them with the crosslinker N,N-(1,2-dihydroxyethylene) bisacrylamide (DHEBA). Afterwards, there was a pre-polymerization step. While a template stamp of influenza virus was prepared, the polymer was spin-coated onto quartz crystal microbalance (QCM) electrodes. The stamp coated with the template virus was then pressed onto the spin-coated pre-polymer and polymerized under 254 nm UV light. At the end, the template was removed from the polymer surface and the virus was denatured in order to possess a final rigid surface to apply on the QCM as a biosensor (Figure 3a). MIPs were made for each influenza viral subtype and each MIP possessed a better recognition property towards its original viral template. Their findings suggested that both the H and N domains played crucial roles in the molecular recognition of the MIP, concluding that the use of influenza A virus MIPs could be a rapid alternative (the sensor signal achieved a fully horizontal response after 3–4 h) to selectively screen different influenza A subtypes in unknown samples [97].

More recently, Ayankojo and co-workers developed an electrochemical sensor for the rapid detection of SARS-CoV-2 protein [98]. The disposable thin-film metal electrodes (Au-TFME) were modified with an MIP film with selectivity for S protein (ncovS1) and the latter was used as the recognition element. The chip was connected to a potentiostat, which measured the ncovS1-specific reduction in the intensity of the charge transfer carried by a redox probe through the MIP film (Figure 3b). The performance of the sensor was studied in both buffer and in COVID-19 patients’ nasopharyngeal swab samples. A remarkable selectivity of the MIP film towards the S protein was achieved by adopting the covalent imprinting approach, i.e., by involving the chemical interaction between 1,2-diol of the highly glycosylated S protein and the boronic acid group of the 3-aminophenylboronic acid (APBA). The developed biosensor was capable of detecting S proteins in untreated saliva with a limit of detection (LOD) of 19 ng/mL and demonstrated a rapid diagnostic possibility with a rebinding time of 15 min and a measurement duration of 5 min, comparable with currently available antigen testing assays. The sensor also demonstrated reasonable discrimination against spike proteins from other variants of the SARS-CoV-2, highlighting the suitability of this diagnostic tool for clinical assessment. Nevertheless, further studies are required to qualify its selectivity against all known strains of the virus and the associated mechanism of selective recognition [98].

A low-cost cotton-tipped electrochemical immunosensor for the detection of SARS-CoV-2 was also developed by Eissa and Zourob [99]. In their work, they combined cotton fibers and electrochemical assays for the detection of the nucleocapsid (N) viral antigen. This was an innovative approach since sample collection and detection tools were integrated into a single platform by coating screen-printed electrodes with absorbent cotton padding. The immunosensor was fabricated by immobilizing the N viral antigen on carbon nanofiber-modified screen-printed electrodes after functionalization of the sensor surface by electrografting (Figure 3c). Carbon nanofibers (CNF) were chosen because of their potential for applications in biosensors, i.e., large surface area, stability, and ease of functionalization. The detection of the viral antigen was achieved via swabbing followed by competition assays using a fixed amount of N protein antibody in solution. The biosensor showed very good sensitivity, with a LOD for the N antigen electrochemical immunosensor of 0.8 pg/mL, and a high selectivity, since it did not show cross-reactivity with antigens from other tested viruses, such as influenza A and HCoV. Additionally, the signal measurements could be made using a handheld potentiostat and easily monitored using a smartphone device (direct and rapid tool for COVID-19 diagnostic) [99].

Regarding IAV, a new and rapid platform for its diagnosis was developed by Park and co-workers [100]. In their work, a conductive polymer [poly(aniline-co-pyrrole)]-encapsulated vesicle (CPV), with a diameter of 218 nm, was combined with a peptide having specificity towards hemagglutinin. CPVs expose distinctive absorbance spectra responding to different distances between CPVs (the strength of π–π interactions is dependent on the distance). In the presence of IAV, the peptide-conjugated CPV (PCPV) specifically bound to the virus, reducing the distance between the CPVs. The consequent agglomeration of PCPV worked as a mechanical stimulation that led to a shifting of π–π interactions and triggered an optical response that facilitated the quantitative detection of the virus. This system not only possessed a sufficient limit of detection (3.37 log10 TCID50/mL), but also a target-responsive signal transduction, being a useful and complementary platform for the rapid detection of IAV [100]. In Table 2, all the examples described above are presented, in order to summarize the possible different tools to detect viruses such as SARS-CoV-2 or influenza A.

## 4. Treatment

Although there is not a cure for COVID-19, several therapeutic strategies have been studied to fight this disease. From RNA polymerase inhibitors (e.g., remdesivir) to guanosine analogs with distinct antiviral mechanisms (e.g., ribavirin), anti-malarial drugs (e.g., chloroquine), convalescent plasma transfusion or antibody-based therapies, [101,102,103] all of them are under research (some in advanced phases of clinical trials) to find a hopeful strategy that ends the pandemic. Despite the potential of these molecules, their delivery to the lungs (where the infection occurs) is still very challenging, and so polymeric nanomedicine strategies may also be a solution for the targeted delivery of these molecules.

Polymer-based nanoparticles are colloidal systems made up of natural or synthetic polymers [104]. Among the natural ones (derived from plant, animal or microbial sources), the most typical examples are hyaluronic acid, albumin, gelatin, chitosan (a derivative of chitin), alginate and collagen, whereas for synthetic polymers, a widely investigated example is polyethylene glycol (PEG) [105]. Hyaluronic acid (HA), generally referred to as hyaluronan, has negatively charged groups (carboxylate groups), highly hydrophilicity and high molecular weight. HA also forms a unique viscous network (viscoelastic properties) [106,107] that makes it an excellent candidate for biomedical applications [108]. For example, Thirumalaisamy and co-workers evaluated the *in silico* antiviral activity of hydrochloroquine (HCQ), a conventional anti-malarial drug with antiviral activity against SARS-CoV-2, and its hyaluronic acid conjugate (HA-HCQ) towards different SARS-CoV-2 protein molecular targets [109]. The HA-HCQ derivative corresponded to HCQ covalently linked through an ester linkage to HA in a specific position (i.e., C5′ carboxylic group of the glucuronic moiety) in order to have an increased bioavailability, safety or controlled released in the body, together with the reduction in its systemic toxicity. The molecular docking study revealed superior binding affinity and interactions of HA-HCQ conjugates towards SARS-CoV-2 molecular target proteins (ranging from −13.2046 KJ/mol to −23.1778 KJ/mol), compared to the free HCQ drug (ranging from −12.2217 KJ/mol to −13.6327 KJ/mol). The HA-HCQ conjugate also showed maximal drug delivery to the respiratory tract with increased drug clearance and less toxicity to host cells. Of course, in vitro and in vivo studies are still needed to confirm not only the drug delivery safety and targeting receptors, but also the toxicity, solubility and efficacy of HA-HCQ conjugates [109].

**Figure 4:**
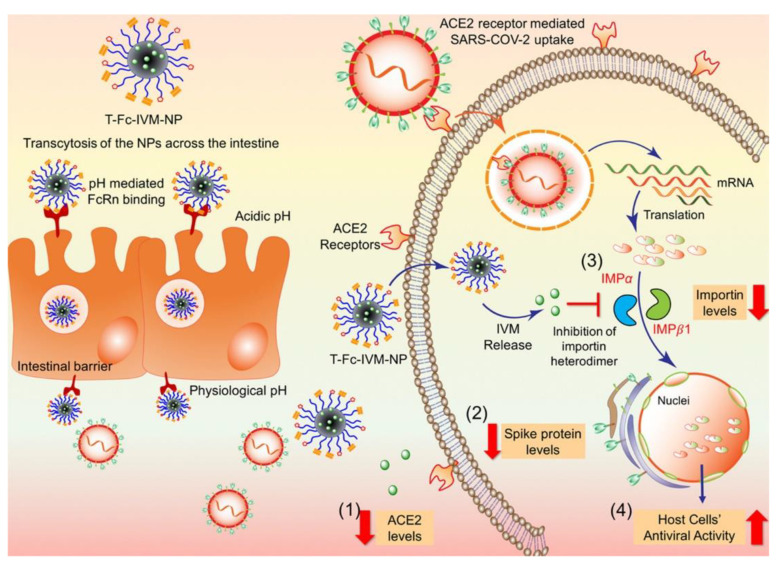
Graphical representation of the targeted-Fc-IVM-NPs in the acidic gut lumen binding FcRn receptors and allowing NPs to transcytose across the intestinal barrier. IVM delivered via T-Fc-IVM-NPs shows the ability to (1) decrease ACE2 receptor levels, (2) decrease SARS-CoV-2 spike protein levels, and (3) decrease levels of the nuclear transport proteins importin α and β1, which leads to (4) an increase in the antiviral activity of infected cells. (Reproduced with permission from [110]. Copyright American Chemical Society, 2020).

Surnar and co-workers developed an orally administrable ivermetacin−NP using poly(lactide-co-glycolide)-*b*-poly-(ethylene glycol)-maleimide (PLGA-*b*-PEG-Mal), a nano-formulation that could allow ivermetacin (IVM) to be gradually released into the bloodstream [110]. IVM is a well-known antiviral drug that has been used for several years to treat many infectious diseases [111]. Recently, an in vitro study showed that IVM was able to inhibit the replication of COVID-19-infected cell lines [112]. Therefore, the developed IVM-loaded NPs, with a size of approximately 70–80 nm, could serve as a nano-vehicle to deliver a more potent therapeutic antiviral dose against COVID-19. In this work, the authors tagged an Fc immunoglobulin fragment to PLGA-*b*-PEG-Mal (T-Fc-IVM-NPs) to take advantage of FcRn-driven crossing of the gut epithelial barrier to reach the bloodstream (Figure 4). The key goal was not to reduce the levels of proteins that contribute to viral infection, but to understand the mechanisms that could prevent viral entry into cells. To test the therapeutic abilities of T-Fc-IVM-NPs against both ACE2 and the viral spike proteins, HEK293T (human embryonic kidney epithelial cells) were transfected with a plasmid containing the SARS-CoV-2 viral spike protein. Subsequently, the cells were treated with IVM or T-Fc-IVM-NPs for a period of 4 h followed by incubation for 20 h. The expression of the spike protein and ACE2 in the HEK293T cells were significantly decreased by the IVM nano-formulation but not by the free IVM. At 4 h of treatment, a differential effect of IVM and IVM nano-formulation was observed and so the NPs were efficient in decreasing the expression of viral S protein and its ACE2 receptor, both of which are keys points to lower disease transmission rates. Nevertheless, the 24 h treatment did not show any difference in IVM and IVM-NPs, which could indicate that IVM-loaded NPs could be able to be taken up into cells earlier than free IVM to exert their effects [110].

A completely different approach was described by Zhang and co-workers, which focused on the affected host cells instead of targeting the causative agent. Having in mind that SARS-CoV-2 binds to protein receptors, either known or unknown, the authors created cellular nanosponges as an effective medical countermeasure to SARS-CoV-2 [113]. The nanosponges were prepared by wrapping polymeric nanoparticle cores, namely biodegradable poly(lactic-co-glycolic) acid (PLGA), with natural cell membranes such as lung epithelial type II cells and human macrophages (cells that are natural targets of SARS-CoV-2). These polymer-based cellular nanosponges, namely epithelial-nanosponge (E-NS) and macrophage-nanosponge (MΦ-NS) showed comparable ability to neutralize the virus with half-maximal inhibitory concentration (IC_50_) values of 827.1 µg/mL and 882.7 µg/mL for E-NS and MΦ-NS, respectively [113]. Nevertheless, for the treatment of COVID-19, MΦ-NS may have major advantages compared to E-NS, since they could not only neutralize the viral activity at an early stage (immune response to the infection), but also at a later stage of the disease (addressing the fulminant inflammation). More recently, some of these authors engineered a cell membrane-coated nanoparticle, with an average diameter of approximately 185 nm, this time to display hemagglutinin (HA), a protein found on IAV surface [114]. The main goal was that the resulting nanocarrier could exhibit virus-mimicking endosomal escape properties and enhanced mRNA delivery to the cytosolic compartment. For that, the membrane from the engineered cells was isolated and coated onto PLGA (HA-mRNA-NP). At 24 h after administration of the nanoparticles, mice were injected with Cypridina luciferin (CLuc) and bioluminescent activity was evaluated using a live animal imaging system. A strong bioluminescence was detected in mice treated with HA-mRNA-NP compared to untreated controls, demonstrating the ability of the engineered HA to promote efficient mRNA delivery in vivo. The same nano-formulations were also evaluated for their ability to elevate the serum levels of a secreted payload after systemic delivery. Mice were intravenously administered with the nanoparticle formulation, and their blood was sampled at 12 and 24 h after injection to monitor CLuc activity. As expected, the untreated control group showed no changes in CLuc signal throughout the study, whereas for the HA-mRNA-NP group, there was a slight increase in bioluminescence at 24 h. Therefore, these results demonstrated that the engineering of cell membrane-coated nanocarriers to express HA can lead to more efficient in vivo mRNA delivery (and increase in protein expression) after both local and systemic administration [114]. In Table 3, there is a short summary of the reported and explored polymer-based strategies for use in the treatment of both SARS-CoV-2 and influenza A.

## 5. Conclusions

This review aimed to appraised works exploring polymeric materials as indispensable tools against RNA viral infections, namely SARS-CoV-2 (COVID-19) and influenza (flu). Looking for three possible hallmarks of these diseases, i.e., protection, detection, and treatment, we saw how versatile (nano)polymeric systems can be.

From face masks to protective clothing or disinfectants, several materials have been explored in this field so that individuals can be protected. The COVID-19 vaccines developed by Pfizer-BioNTech and Spikevax were also discussed as a successful protective approach to tackle viral-related complications. However, they have in their formulation polyethylene-glycol (PEG). Besides being a synthetic polymer, it is also associated with allergic reactions, and so we gave emphasis to new alternatives using natural polymers or their derivatives (e.g., chitosan, gellan gum and carrageenan).

In the past two years, due to the pandemic, we have heard a lot of RT-PCR-based assays as primary diagnostic tools for SARS-CoV-2 detection. However, these methods are expensive and have high detection times (among other issues). Polymeric nanosensing devices, namely MIPs or immunosensors, offer a more versatile, rapid, and reliable technique to detect coronavirus or influenza A.

Regarding treatment strategies, different polymer-based nanotechnologies have been successfully developed worldwide. Great examples have been explored in the inhibition of SARS-CoV-2 infection, such as the orally administrable Ivermetacin-loaded NPs or cellular nanosponges. In both cases, synthetic polymers were used, namely poly(lactide-co-glycolide) (PLGA). Once again, to choose biopolymers over synthetic ones represents the next step for the development of more sustainable materials. This may epitomize the future perspectives in this field, where it is aimed to develop greener solutions for viral protection/detection/treatment. In the end, all of the reported developments represent novel solutions to battle viral infections, especially against COVID-19 that is still ongoing. The next step is to understand what is still missing in these solutions to reach the market and what may be the related problems. Doubtlessly, mixing polymers and nanotechnology can result in a strong tool to fight respiratory viruses and their associated diseases.

## Figures and Tables

**Table 1:** Polymer-based strategies applied in the prevention of SARS-CoV-2 and influenza A viruses.

Strategies	Virus	Type of Polymer	Results	Ref.
Face masks	SARS-CoV-2	Synthetic (polypropylene, polyurethane, polyacrylonitrile, polystyrene)	Effective in viral inactivation but associated with environmental pollution (affecting both human and animal health).	[40,41,42,43]
		Natural (cellulose, alginate)	The BCNWs inactivated >99% of the viruses, while the fabrics coated with alginate–Cu(II) presented a 99% viral inactivation when in contact with the virus for less than 1 min.	[51]
	Influenza A	Natural (cellulose)	The filters proved to not only to block the droplet-borne/airborne IAV, but also to remove 99.999% of a sprayed solution of T4D bacteriophages after 1 h.	[53]
	L. innocua *	Synthetic (polyacrylonitrile and poly(vinylalcohol-co-ethylene))	The nanofibers membranes showed a high antiviral efficiency of >99.9% within a short exposure time (<90 min).	[49]
Protective clothing	SARS-CoV-2 Influenza A	Synthetic (poly(methyl methacrylate))	The PMMA nanofibers showed high performance as an antiviral agent.	[46]
	SARS-CoV-2	Synthetic (polyvinyl alcohol)	With a 3% concentration of AV, the antibacterial activity of the nanofibers was excellent, with high zone of inhibition values of 10.50 (trial 1), 10.79 (trial 2) and 11.08 mm (trial 3).	[47]
Disinfectants	MERS-CoV	Natural (cellulose)	Anti-viral inhibitory effect of 48.3%.	[65]
	Influenza A	Synthetic (polyoxyethylene–polyoxypropylene)	In a short period of time (1 min), IAV was killed.	[66]
Vaccines	SARS-CoV-2	Synthetic (polyethylene-glycol)	Effective, but associated with allergic reactions.	[68,69]
		Natural or derivative(chitosan)	On day 45 after intranasal immunization, the mucosal, systemic humoral and cell-mediated immune responses were highly stimulated (with a robust production of immunoglobulins or CD8^+^ cells).	[78]
	Influenza A	Natural or derivative (N,N,N-trimethyl chitosan)	Optimal immune response with the production of IL-1β (by macrophages) at 16 mg/mL and IL-2 (by lymphocytes) at 64 mg/mL.	[81]
		Natural (alginate)	On day 7 post challenge, the frequency of lung CD8+ cells was 26 ± 2.7% in mice that received the polymer encapsulated vaccine, comparing to mice injected with alginate alone (1.4 ± 0.07%) or non-vaccinated mice (2 ± 0.6%).	[84]
Nasal spray	SARS-CoV-2	Natural (gellan gum and carrageenan)	Potent antiviral spray (in a proportion of 75:25, gellan to λ-carrageenan) with protective and inhibitory effects against SARS-CoV-2 (suppression of the infection up to a dilution of 1/100 in comparison with the untreated control group).	[85]
		Natural (hydroxypropyl cellulose and solagum)	At 5% concentration, the final Covispray composition neutralized nearly 30% of IL-6, 80% of TNF-α and GM-CSF, as well as more than 90% of pro-inflammatory cytokines.	[89]

BCNWs: biocidal cellulose nonwovens; IAV: influenza A virus; AV: aloe vera; GM-CSF: granulocyte-macrophage colony-stimulating factor; PMMA: poly(methyl methacrylate); IL: interleukin; TNF-α: tumor necrosis factor-α. * Although not directly related to SARS-CoV-2 or influenza A, this study may have hopeful applicability to COVID-19 or flu. Further assays are needed to understand the potential of these membranes as inhibitors towards these viruses.

**Table 2:** Polymer-based strategies applied in the detection of SARS-CoV-2 and influenza A viruses.

Strategies	Virus	Type of Polymer	Results	Ref.
MIPs	SARS-CoV-2	Synthetic (poly-3-aminophenylboronic acid)	The biosensor demonstrated a rebinding time of 15 min and a measurement duration of 5 min, being comparable with the current available antigen testing assays.	[98]
	Influenza A	Synthetic (polyacrylamide, poly-methacrylic acid, poly-methylmethacrylate and poly-N-vinylpyrrolidone)	Each MIP possessed a better recognition property towards its original viral template. A fully horizontal response was obtained after 3–4 h.	[97]
Electrochemical immunosensor	SARS-CoV-2	Natural (cotton fibers)	The biosensor showed a very good sensitivity, with a LOD of 0.8 pg/mL, and also a high selectivity, since it did not show cross-reactivity with antigens from other tested viruses.	[99]
Diagnosis platform	Influenza A	Synthetic (poly(aniline-co-pyrrole))	The detection system possesses a sufficient level of LOD (3.37 log10 TCID_50_/mL) and a target-responsive signal transduction.	[100]

MIP: molecularly imprinted polymer; LOD: limit of detection; TCID_50_: median tissue culture infectious dose.

**Table 3:** Polymer-based strategies applied in the possible treatment of SARS-CoV-2 and influenza A viruses.

Strategies	Virus	Type of Polymer	Results	Study Type	Ref.
Drug-polymer conjugate	SARS-CoV-2	Natural (Hyaluronic acid)	Superior binding affinity of the conjugates (ranging from −13.2046 KJ/mol to −23.1778 KJ/mol), comparing to free HCQ drug (ranging from −12.2217 KJ/mol to −13.6327 KJ/mol).	*In silico*	[109]
Loaded NPs	SARS-CoV-2	Synthetic (PLGA-PEG-Mal)	At 4 h treatment, the IVM-NP was able to decrease the expression of viral spike protein and its receptor angiotensin-converting enzyme 2.	*In vitro* *In vivo*	[110]
	Influenza A	Natural (Alginate)	Strong humoral and cellular immune response with the activation of B and dendritic cells, as well as secretion of cytokines.	*In vitro* *In vivo*	[83]
Cellular nanosponges	SARS-CoV-2	Synthetic (PLGA)	The cell membranes showed comparable ability to neutralize the virus with IC_50_ values of 827.1 µg/mL and 882.7 µg/mL for E-NS and MΦ-NS, respectively. Neutralization of the virus occurs in a concentration-dependent manner.	*In vitro* *In vivo*	[113]
	Influenza A	Synthetic (PLGA)	The *in vitro* activity resulted on a successful expression of hemagglutinin. The *in vivo* activity showed a significant increase in protein expression (increase in bioluminescence at 24 h) in both local and systemic delivery scenarios.	*In vitro* *In vivo*	[114]

PLGA-PEG-MAL: poly(lactide-co-glycolide)-poly-(ethylene glycol)-maleimide; PLGA: poly(lactide-co-glycolide); HCQ: hydrochloroquine; IVM-NP: ivermetacin nanoparticle; E-NS: epithelial-nanosponge; MΦ-NS: macrophage-nanosponge.

## Data Availability

Not applicable.
